# Quantitative trait loci-dependent analysis of a gene co-expression network associated with Fusarium head blight resistance in bread wheat (*Triticum aestivum* L.)

**DOI:** 10.1186/1471-2164-14-728

**Published:** 2013-10-24

**Authors:** Karl G Kugler, Gerald Siegwart, Thomas Nussbaumer, Christian Ametz, Manuel Spannagl, Barbara Steiner, Marc Lemmens, Klaus FX Mayer, Hermann Buerstmayr, Wolfgang Schweiger

**Affiliations:** 1 Munich Information Center for Protein Sequences/Institute for Bioinformatics and Systems Biology, Helmholtz Center Munich, D-85764 Neuherberg, Germany; 2 Institute for Biotechnology in Plant Production, IFA-Tulln, University of Natural Resources and Life Sciences, A-3430 Tulln, Austria

**Keywords:** *Triticum aestivum*, Bread wheat, *Fusarium graminearum*, Fusarium head blight, *Fhb1*, *Qfhs.ifa-5A*, Transcriptome, Gene co-expression network, RNA-seq

## Abstract

**Background:**

Fusarium head blight (FHB) caused by *Fusarium graminearum* Schwabe is one of the most prevalent diseases of wheat (*Triticum aestivum* L*.*) and other small grain cereals. Resistance against the fungus is quantitative and more than 100 quantitative trait loci (QTL) have been described. Two well-validated and highly reproducible QTL, *Fhb1* and *Qfhs.ifa-5A* have been widely investigated, but to date the underlying genes have not been identified.

**Results:**

We have investigated a gene co-expression network activated in response to *F. graminearum* using RNA-seq data from near-isogenic lines, harboring either the resistant or the susceptible allele for *Fhb1* and *Qfhs.ifa-5A*. The network identified pathogen-responsive modules, which were enriched for differentially expressed genes between genotypes or different time points after inoculation with the pathogen. Central gene analysis identified transcripts associated with either QTL within the network. Moreover, we present a detailed gene expression analysis of four gene families (glucanases, NBS-LRR, WRKY transcription factors and UDP-glycosyltransferases), which take prominent roles in the pathogen response.

**Conclusions:**

A combination of a network-driven approach and differential gene expression analysis identified genes and pathways associated with *Fhb1* and *Qfhs.ifa-5A*. We find G-protein coupled receptor kinases and biosynthesis genes for jasmonate and ethylene earlier induced for *Fhb1*. Similarly, we find genes involved in the biosynthesis and metabolism of riboflavin more abundant for *Qfhs.ifa-5A*.

## Background

Bread wheat (*Triticum aestivum* L.) is one of the most important food crops worldwide. 20% of the human calorie and protein uptake derive from wheat. One of its most prevalent and destructive pathogens is the fungus *Fusarium graminearum* (teleomorph *Gibberella zeae,* Schwabe) [1,2]. *F. graminearum* frequently infects wheat and other small grain cereals in temperate regions throughout the world. Especially under humid weather conditions spores that have overwintered in remaining plant debris on the field reach the flowering wheat head via splash water, from where the germinating fungus penetrates the more susceptible floral tissue. The resulting disease Fusarium head blight (FHB) annually accounts for severe losses in grain yield and also quality due to the contamination with mycotoxins produced by the fungus. Among these deoxynivalenol (DON) holds a key position. DON is a potent inhibitor of protein biosynthesis and constitutes a serious threat to human and animal health in food and feed [3]. This has, among others, prompted the European Union to enact maximum tolerated levels in food [4] and advisory levels were issued by the Food and Drug Administration in the United States. Consequently, developing high yielding and *F. graminearum* resistant varieties is of high priority for breeders. Despite its economic relevance, the genomic sequence of wheat is not yet available due to its sheer size (~ 17 Gb) and its highly repetitive nature [5,6]. However, a large body of publications aiming towards genetic mapping of resistance genes against *F. graminearum* has been published in the last 14 years and so far over 100 quantitative trait loci (QTL) have been described to contribute to resistance [7]. Two highly reproducible and large-effect QTL are *Fhb1* located on the short arm of chromosome 3B [8] and *Qfhs.ifa-5A* on chromosome 5A [9]. Depending on the genetic background both reduce disease symptoms by 20–25% and confer either type II resistance against spreading of the disease (*Fhb1*) or type I resistance against initial penetration (*Qfhs.ifa-5A*) [9,10]. *Fhb1* was linked to the higher ability to enzymatically inactivate DON by glycosylation [11], but recent reports also associate the higher formation of phenylpropanoids [12] or a non-responsive susceptibility factor, *WFhb1_c1*[13] with the activity of the QTL. No functional evidence has been proposed for *Qfhs.ifa-5A*. A donor of both QTL is the CIMMYT (http://www.cimmyt.org) derived line CM-82036, a progeny of the prominent resistance source Sumai-3. CM-82036 also encodes for multiple minor effect resistance QTL, which provide the line with a significantly higher level of resistance when compared to a near-isogenic line stacking both *Fhb1* and *Qfhs.ifa-5A* resistance alleles in a susceptible background [10,14]*.* Recent years have seen multiple transcriptomic and proteomic studies investigating the *F. graminearum*/wheat-interaction, which have helped developing an understanding of the general response against the fungus (reviewed in [2]), but these did not lead to the identification of QTL-related resistance genes so far.

RNA-sequencing technology is well established as an alternative to microarrays. The major obstacle for the analysis of the entire wheat transcriptome is the availability of a suitable mapping reference covering the gene space of the yet unsequenced species. Establishing the gene-space as a reference is even more challenging as the three homeologous genomes of polyploid bread wheat share a high level of sequence similarity. A recent study [15] tried to overcome these limitations by combining short reads from Illumina technology with *454* data in a two-stage assembly. The TriFLDB [16] database collected available full-length coding sequences from wheat over the last years. Currently, efforts are underway to assemble the wheat genome entirely using chromosome arm sorting [17], genotyping by sequencing [18] and whole genome profiling approaches [19]. The most complete assembly of the *T. aestivum* gene space is described by the released wheat low-copy-number genome (LCG) assembly [5], generated from *454* sequences and reference as well as progenitor genomes, which provides partial sequences of an estimated number of 94 - 96 k genes. In addition, the transcriptome of the close relative barley (*Hordeum vulgare* L.) comprising more than 26 k genes has been annotated on a WGS assembly and anchored to the physical map [20]. These data, the wheat LCG assembly and the homology to the complete barley gene space provide a novel and unique reference for RNA-profiling studies, allowing a high specificity and coverage of the transcriptome.

To gain novel insights into the defense response of wheat against *F. graminearum* using these newly available data resources, we have sequenced the transcriptome of five differently resistant genotypes, comprising a set of four near-isogenic lines (NILs) harboring either, both or none of the resistance alleles of *Fhb1* and *Qhfs.ifa-5A* in the susceptible background of the German spring wheat cultivar Remus and the highly resistant QTL-donor line CM-82036. While most of the existing, microarray-based analyses aimed at analyzing single genes, we here provide a transcriptome-wide approach and focus on investigating the interaction of genes. Several studies have demonstrated the power of co-expression networks for detecting groups of genes that react in a coordinated effort against pathogen response, e.g. in cucumber and rice [21,22]. In the present study we introduce a network-driven approach, which led to the identification of groups of genes that form functional clusters of co-expressed genes. Additionally, we screened for single genes that occupy central positions within the newly established network (e.g. hub genes). We find that these putative key genes in the response to *F. graminearum* are members of prominent pathogenesis-related gene families. We further investigate differential expression patterns observed for the glucanase, nucleotide-binding site leucine-rich repeat (NBS-LRR), WRKY and UDP-glycosyltransferase (UGT) gene families, which hold relevant positions in our analysis.

## Results

### Data harvesting, processing and quality control

We extracted RNA from spike tissue of five different wheat genotypes that were treated with a *F. graminearum* spore suspension or mock 30 and 50 hours after inoculation (hai). All lines showed distinct levels of resistance after point inoculation in green house trials [10,14]. The lines comprised a set of four NILs that harbor either of the *F. graminearum*-resistance QTL *Fhb1* (NIL2, moderately resistant) or *Qfhs.ifa-5A* (NIL3, moderately resistant), both of these QTL (NIL1, resistant) or none of them (NIL4, susceptible) in the genetic background of the *F. graminearum* susceptible German spring wheat cultivar Remus. These lines are at least 96% isogenic as shown with DArT markers [14], but do contain QTL-unrelated, yet linked genes from the original QTL donor in the introgressed section. Additional samples were collected from the highly resistant QTL-donor line CM-82036, which encodes in addition to *Fhb1* and *Qfhs.ifa-5A* for multiple minor-effect QTL. Samples were sequenced on an Illumina HiSeq2000 platform, which summed up to a total of 1,827 Gb raw sequences (Additional file 1). RNA-Seq reads were compared against public wheat full-length cDNA [16] to ensure the quality and coverage of genes along the entire length (Additional file 2). This allowed to map reads on the LCG assembly [5] resulting in 233,780 Cuffmerge transcripts, out of which 151,853 (65%) transcripts are expressed in all five genotypes (Table 1). To assess the progress of the disease, reads were compared to the *F. graminearum* transcriptome [23]. In average 87 k reads (0.3% from the average of total reads) were matching *F. graminearum* genes for samples inoculated with spore suspensions and no more than about 1.8 k reads in the mock-treated samples (Additional file 3). This observation can be explained by contaminations, mapping errors or conserved domains. One particular mock treated sample (NIL2, 50 hai, replicate 3) contained an unexpected high number of reads (10.7 k reads) that matched *F. graminearum* genes and was therefore excluded from further analysis. While samples taken at 50 hai showed in general a higher abundance of *F. graminearum*-mapped reads than 30 hai samples, we could not detect significant differences between the infected lines at any time point (Additional file 4).

**Table 1:** Mapping of RNA-seq data

**Line**	**Cufflinks**	**Barley BBH**	**DEG (barley BBH)**
**CM-82036**	183,540	15,360	1,956 (13%)
**NIL1**	189,486	15,797	1,781 (11%)
**NIL2**	196,078	15,734	2,067 (13%)
**NIL3**	192,584	15,676	1,954 (12%)
**NIL4**	186,755	15,680	2,005 (13%)

Overview of generated cufflinks genes with the corresponding number of barley best bi-directional hits (BBH) as well as the number of differentially expressed genes (DEG) is given. Overall 233,780 cufflinks genes were found in all five lines, with 151,853 genes in common.

Cuffdiff [24] was used to extract differentially expressed genes (Benjamini-Hochberg correction [25] (BH); *p* < 0.1; see Methods). As the LCG contigs are in general short (average length: 714 bp) and represent only partial genes due to the high (genic) sequence redundancy in hexaploid wheat, we used the recently published barley high confidence genes (*N* = 26,159, [18]) to filter the previously generated fragmented Cufflinks transcripts for gene candidates. Thereby, with the barley best bidirectional hit (BBH), we were able to link 16,964 transcripts to barley genes. Mapping to barley homologs has two major impacts: It drastically reduces the number of analyzed transcripts in the analysis and also wheat-specific genes with no barley homologs may be lost. Moreover, a differentiation between homeologous genes from different genomes is not always possible. However, the remaining transcripts have a higher quality in terms of gene calling confidence. Additionally, for these data trustworthy sets of Gene Ontology (GO) and Interpro annotations exist for analyzing the remaining transcripts. Overall these sets comprised 9,776 genes with GO annotations and 14,164 genes with Interpro annotations. Therefore, we performed the subsequent enrichment analyses and network inference on the barley BBH reduced subset.

### Genotype-specific differentially expressed genes link *Fhb1* to early induction of jasmonate and ethylene biosynthesis and *Qfhs.ifa-5A* to riboflavin biosynthesis and lipid binding

With Cuffcompare 233 k cufflinks genes were combined and tested for differential expression by comparing *F. graminearum* inoculated samples with the respective mock-treated sample (FDR adjusted *p-*value < 0.1). Per genotype between 183,540 (CM-82036) to 196,078 (NIL2) Cufflinks genes were assembled. BBH assigned barley genes were found for 15,360 (CM-82036) to 15,797 (NIL1) Cufflinks genes. In average 8% of those genes were differentially expressed, for Cufflinks genes with BBH linkage 11-13%. To provide a more granular insight into differentially expressed genes, we analyzed common differentially expressed genes (DEG) for 30 hai and 50 hai separately. A list of all analyzed differentially expressed genes is provided in Additional file 5.

**Figure 1:**
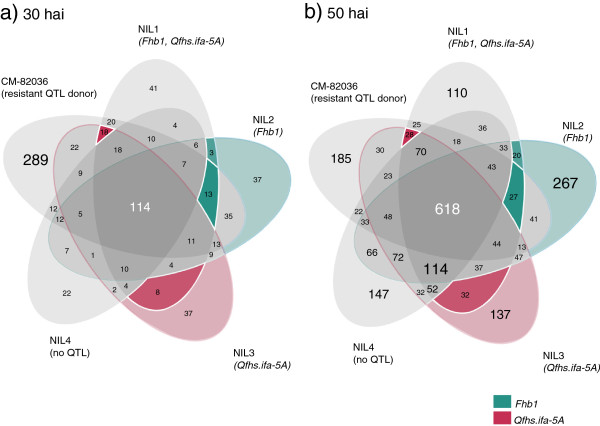
**Differentially expressed genes per line.** Venn diagrams showing unique *F. graminearum* responsive genes at 30 hours after inoculation (hai) **(a)** and 50 hai **(b)** for the investigated genotypes (CM-82036, NIL1 (harboring both resistance QTL, *Fhb1* and *Qfhs.ifa-5A)*, NIL2 (*Fhb1*), NIL3 (*Qhfs.ifa-5A*) and NIL4 (no QTL)) as well as genes shared between them in the respective intersections. Intersections of lines sharing either of the two QTL harbor genes associated with these QTL. These are highlighted in cyan (*Fhb1*) or magenta (*Qfhs.ifa-5A*).

The earlier sampling time point 30 hai was characterized by a pronounced response of the resistant CM-82036 (Figure 1a). From the 1,302 DEG identified in total, 289 were differentially expressed only for CM-82036 compared to 114 DEG, which were shared between all five genotypes. The group of DEG shared only between NILs 1–4 and also the number of DEG specific for each NIL comprised only few genes (10 to 41). 50 hai a large part (618) of the 2,470 DEG was significantly changed for all genotypes. At this time point, in contrast to 30 hai, all genotypes exhibited a similarly strong response in terms of DEGs (Figure 1b). Also a large group of DEG was shared between the four NILs (114) representing the response of genes in the genetic background of the susceptible recurrent parent Remus.

GO terms obtained via topGO [26] for these contrasts represented genotype dependent defense responses (Additional file 6 for 30 hai and Additional file 7 for 50 hai). Regardless of QTL all genotypes shared essential pathogenesis associated pathways at 30 hai: These included the biosynthesis of phenylpropanoids and polyamins and also genes involved in the reduction of oxidative stress and chitinases. We also found a glutamate synthase more abundant 30 hai and an amino acid transporter more abundant at 50 hai. Both may be linked to multiple pathogen-induced reactions in the primary and secondary metabolism [27,28]. Moreover, an UDP-N-acetylmuramate dehydrogenase was also upregulated 30 hai, which potentially acts in biosynthesis of amino sugars used for posttranslational protein modification. 50 hai we observed additional terms related to ubiquitination and the biosynthesis of tryptophan.

The response unique for CM-82036 comprised a high number of terms corresponding to signaling events and transcription factors at the early time point and also terms corresponding to the biosynthesis of trehalose and terpenoids. The response at 50 hai included UGTs, cytochrome P450 monooxygenases (CYP) and terms related to the primary metabolism involved in amino acid biosynthesis and gluconeogenesis.

Genes associated with the activity of *Fhb1* or *Qfhs.ifa-5A* should be represented by DEG shared by NILs harboring these QTL (highlighted sections in Figure 1). In the section shared by NIL2 (resistant allele of *Fhb1*) and NIL1 (both QTL) and in the section shared by both NILs and CM-82036 we identified 16 genes collectively at 30 hai and 47 at 50 hai. Similarly, 26 and 60 genes were shared in lines harboring the resistance allele of *Qfhs.ifa-5A* (NIL1 and NIL3 containing *Qfhs.ifa-5A* only and optionally CM-82036). We also looked at the differentially expressed genes unique for the genotypes harboring only either of both QTL (NIL2 and NIL3), as the activity of QTL-related genes might not be similarly significantly changed at the observed time point in all lines harboring these QTL due to the different resistance levels.

The specific response of the NIL2 containing *Fhb1* was characterized by the early upregulation of transcription factors and biosynthesis genes for jasmonic acid (JA) and ethylene (ET). Both signaling molecules regulate defense responses in plants against biotic stresses. At 50 hai we found terms related to translation, protein folding and ribosomal protein more abundant. For transcripts shared between lines with *Fhb1* we identified GO terms relating to protein secretion and signal transduction (G protein-related) at 30 hai and terms related to the metabolism of glutamine at 50 hai. Lines containing *Qfhs.ifa-5A* (NIL1 and NIL3) showed higher abundance of gene transcripts related to the tryptophan biosynthesis pathway already at 30 hai and for genes related to lipid binding at 50 hai. GO terms identified in the shared sections are involved in riboflavin production and ET biosynthesis (30 hai). We also found a transcript encoding a glutamate-gated ion channel (30 hai), which controls Ca^2+^-influx into the cell. Similarly to *Fhb1,* these sections also included terms for ribosome biogenesis and protein translation.

### Gene co-expression network analysis identifies defense-associated modules

**Figure 2:**
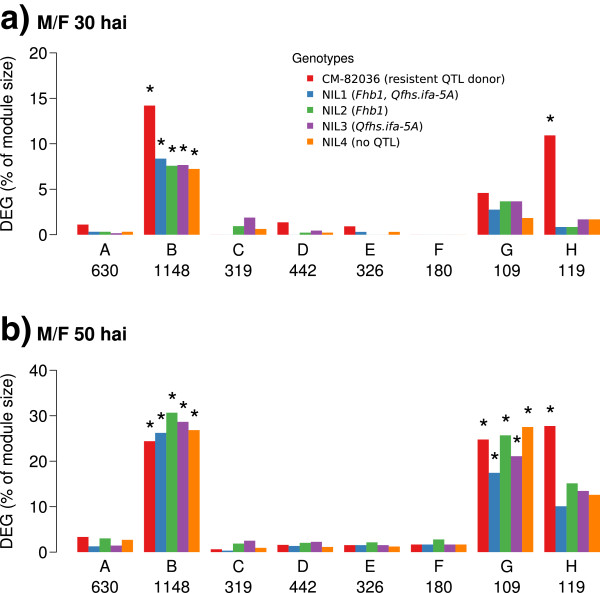
**Differentially expressed genes per module.** The bar plots indicate the ratio of *F. graminearum* responsive differentially expressed genes (DEG) per network module for 30 hours after inoculation (hai) **(a)** and 50 hai **(b)**. To test whether the number of DEG genes was significantly higher than expected by chance we applied a one-sided Fisher’s exact test. Stars indicate a significant enrichment at a Bonferroni adjusted *p*-value smaller than 0.05.

We analyzed the co-expression data from the barley-mapped transcripts of all samples to infer a gene co-expression network specific for the observed conditions. In contrast to the detection of single DEG, this approach takes into account all 20 experimental conditions (covered by 59 samples) simultaneously and allows detecting groups of genes that show similar expression patterns in an untargeted approach. The resulting network contained 3,412 genes after filtering using the coefficient of variation. The co-expressions of these genes were then fitted against a power-law model using the WGCNA package in R [29]. We extracted eight modules (designated module A to module H) from our network, each represented by a group of genes that share similar expression patterns (Figure 2; Additional file 8). Module sizes ranged from 109 (module G) to 1,148 genes (module B), while 139 genes could not be assigned to any module. (Additional file 9).

By using the module eigengenes [30,31] we found that modules B, G, and H were strongly linked to *F. graminearum*-inoculated samples (Additional file 10). Of these modules two exhibited a general association to all genotypes at either both time points (module B) or only 50 hai (module G). Module H was strongly linked to the specific defense response of CM-82036 at 50 hai. Module A was also specific for CM-82036, but not for treatment or time point. A one-sided Fisher’s exact test (significance threshold for Bonferroni adjusted *p-value*s set to 0.05) was applied to test whether these modules showed a higher ratio of DEG than expected by chance. At 30 hai module B was strongly enriched for DEG for all five lines (Figure 2a) with CM-82036 exhibiting the highest relative amount of DEG (*p =* 7.0 e-48). This changed at 50 hai where all four NILs show a higher level of enrichment compared to CM-82036 (maximum *p =* 3.6 e-65; Figure 2b). Also, 50 hai all lines exhibited a higher ratio of DEG for module G (maximum *p =* 1.7 e-02). Module H showed enrichment for CM-82036 at 30 hai (*p* = 1.9 e-02) as well as 50 hai (*p =* 2.7 e-07). We analyzed these data with GO and Interpro terms to obtain functional annotations for the modules. Among others, DEG in the *F. graminearum* responsive module B encoded glutathione S-transferases (GST), UGTs, glucanases, protein kinases and WRKY transcription factors (Additional file 11). For the CM-82036 related Module H and also for module G the few available GO terms did not provide sufficient meaningful annotations to predict specific molecular functions (Additional file 11).

Since module B is the by far largest module and highly enriched for *F. graminearum* responsive genes across all five lines, we further analyzed this module by splitting it into smaller submodules (deepsplit = 4; minimum module size = 10). The two largest submodules comprised 475 (B-sub1) and 397 genes (B-sub2), respectively. Submodule B-sub1 was significantly enriched for DEG in all genotypes at 50 hai but only few DEG (between 5% and 10% of module size) were identified at 30 hai (Additional file 12). The relatively highest amount of DEG was found for the susceptible NIL4 and the moderately resistant NIL2. Only few GO terms were identified for this submodule (Additional file 13). B-sub2 showed a strong enrichment for DEG at 30 hai for all genotypes (minimal *p =* 2.8 e-07). This enrichment was slightly more pronounced for CM-82036, NIL1 and NIL3. These three genotypes share the resistant allele of *Qfhs.ifa-5A*. Consequently, B-sub2 may be associated to the activity of *Qfhs.ifa-5A*. The majority of GO terms for DEG in this submodule were similar to the terms identified for the pool of DEG shared by genotypes harboring *Qfhs.ifa-5A* (see previous section). These corresponded to kinase activity, glutamate-gated ion channels and tRNA aminoacylation (Additional file 13).

### Defense-related central genes in the co-expression network

A gene network allows quantifying the relative importance of single genes (nodes) by making use of local centrality measures [32-34]. Multiple methods exist for assessing the centrality of nodes. Here we applied two methods for ranking the genes by their relative importance within the network: The degree centrality ranks nodes by the number of adjacent nodes within the network, which allows selecting so called hub genes. These hub genes often play important roles in the regulation of gene expression and may provide valuable insight into stress response or genome evolution [35-37]. For our analysis we applied a weighted version of this measure as implemented in the igraph package [38]. Additionally, we also made use of the eigenvector centrality [39], which is related to eigenvectors of the largest eigenvalue of the adjacency matrix. To filter for the most important nodes from these two measures we used the 90% percentile and deemed nodes with values higher than this threshold as being central within the network. We will further refer to degree centrality selected genes as DCG, and to Eigenvector centrality derived genes as ECG.

In our network 218 central genes (ECG + DCG) were significantly regulated after *F. graminearum* inoculation and thus hold prominent roles in the wheat response to the pathogen. These central genes were also more likely differentially expressed in response to the pathogen than non-central genes in the network (Fisher’s Exact Test, BH-adjusted *p* < 0.05). Most belong to module B and only few were identified in other modules. These genes were highly enriched for GO terms associated with signaling, ubiquitination, hypersensitive response and ATP binding. The latter two are GO terms commonly used to describe NBS-LRR resistance genes, which play crucial roles in pathogen reception and signal transduction. Additional terms corresponded to nucleotide binding, suggesting the involvement of transcription factors (including WRKY, for which we found also terms in module B). Interpro annotations further identified GSTs, CYPs, glucanases and UGTs (Additional file 14). Both DCG and ECG are highly connected to other genes. Their expression behavior may have a strong impact on the global expression pattern within the network. When looking at genotype specific changes in expression of central genes, we found a group of 34 genes that were earlier differentially expressed for CM-82036 compared to the NILs. Three central genes, for which we could not retrieve annotations, were only transiently expressed. They were significantly changed for 30 hai but not 50 hai for CM-86036, while in the NILs these genes were differentially expressed only 50 hai. Yet, we detected no central genes that were only changed for CM-82036 but not for the NILs. On the other hand, 35 genes were differentially expressed exclusively for the NILs (Additional file 15). When regarding genotypes differing in the presence of either QTL, we found five central genes earlier induced for *Qfhs.ifa-5A*: These encode four protein kinases and a CYP. Three of these are also present in the *Qhfs.ifa-5A*-associated submodule B-sub2. One of these genes XLOC_099598 encoding a protein kinase ranked third within the DCG, making it one of the highest connected central genes in our network. Similarly, we identified a UGT, an NBS-LRR and a putative disease resistance gene as earlier induced in lines containing *Fhb1*.

### Gene family specific differential expression profiles

The arms race between plants and pathogens has led to the rapid evolution of genes involved in the interaction with the pathogen and consequently to an increase in copy numbers to form large gene families. This allows plants to adapt to new challenges or to overcome detrimental effects of random mutagenesis by redundant gene function. The present study has among others identified glucanases, NBS-LRR proteins, WRKY transcription factors and UGTs as relevant factors in the *F. graminearum*/wheat interaction – each representing a certain stage in the host defense response (recognition, signal transduction, defense regulation and toxin inactivation). To further elucidate genotype and time point specific abundance of such transcripts we expanded our analysis by taking into account the entire gene families.

We extracted Cufflinks genes encoding glucanases, NBS-LRR proteins, WRKYs and UGT using either domain specific motifs or homology information, clustered the acquired sequences using CLUSTALX N-J bootstrapping [40] and added genotype-specific DEG information (Table 2). Using this approach we identified 568 putative wheat glucanase genes via mapping against barley genes that contained the Interpro domain IPR008985. Given the hexaploid nature of wheat, a reasonable high number compared to the 262 putative glucanases in barley [20]. Similarly, we identified 246 NBS-LRR genes via mapping against 267 barley genes (http://www.vmatch.de), 116 WRKY transcription factors (74 in barley) via mapping against a conserved motif (WRKYGQK) and 222 putative UGTs (159 predicted functional genes in *Brachypodium distachyon* Beauv., [41]) by searching for the conserved signature motifs [42,43].

**Table 2:** Differential expression of pathogen-induced gene families


**30 hai**									
	**Total**	**CM-82036**	**NIL1**	**NIL2**	**NIL3**	**NIL4**	**all lines**	**CM-82036 only**	**NILs only**
**glucanases**	112	83	40	41	48	35	13	37	4
**UGTs**	36	31	15	13	20	15	7	13	2
**WRKYs**	21	14	10	3	5	6	1	8	1
**NBS-LRR**	44	36	8	9	11	7	2	26	0
**50 hai**									
	**Total**	**CM-82036**	**NIL1**	**NIL2**	**NIL3**	**NIL4**	**all lines**	**CM-82036 only**	**NILs only**
**glucanases**	304	178	184	214	190	179	91	23	27
**UGTs**	79	27	17	24	21	19	10	7	0
**WRKYs**	44	23	19	23	19	17	5	8	1
**NBS-LRR**	144	83	76	86	86	76	34	13	8

Genotype specific differential expression of genes from selected gene families at 30 hours after inoculation with *F. graminearum* (hai) and 50 hai. Column ‘total’ describes the total number of differentially expressed genes at a certain time point. ”All lines” refers to genes that were differentially expressed for all five lines.

The DEG profiles for the NBS-LRR and glucanase genes showed dramatic differences between CM-82036 and the NILs at 30 hai: 36 of 44 differentially expressed NBS-LRR genes and 83 of 112 differentially expressed glucanases were found changed for CM-82036 and about half of those genes were only changed for this genotype and not for the NILs. In contrast only 7 to 11 of the NBS-LRR and 35 to 48 of the glucanases were differentially expressed for the NILs. 50 hai all genotypes showed an equally high number of upregulated NBS-LRR and glucanase genes (up to 35% of the total number of identified NBS-LRR and glucanase genes). The dominance in gene numbers and genotype specific genes for CM-82036 at 30 hai was not observed at 50 hai. Neither for glucanases (Additional file 16), nor for NBS-LRR (Additional file 17) we observed differential expression patterns that would suggest an *Fhb1-* or *Qfhs.ifa-5A*-dependent upregulation of genes.

**Figure 3:**
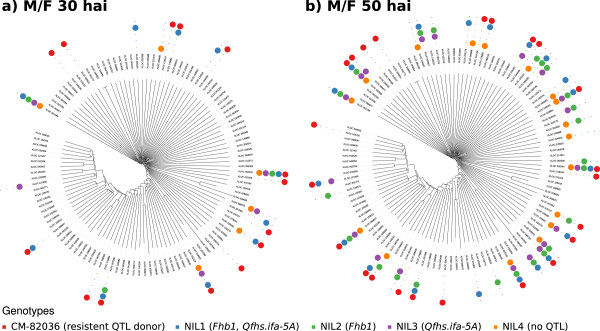
**Regulation of WRKY genes at different time points.** Dendrograms display differential expression of WRKY genes **(a)** 30 and **(b)** 50 hours after inoculation (hai) with *F. graminearum* spores. Genes that are significantly changed for the given genetic background (NIL1-NIL4, CM-82036) in response to *F. graminearum* are indicated in the respective color. Note that the clades within dendrogram do not necessarily reflect groups or families of related genes, but are only used for presentation purposes.

Members of the WRKY transcription factors play a decisive role in regulating response to abiotic and biotic stresses [42]. While CM-82036 showed a stronger response at 30 hai (14 genes), we also found a relatively high number (10) of WRKY upregulated for the more the resistant NIL1 (containing *Fhb1* and *Qfhs-ifa.5A*) compared to the moderately to susceptible NILs (NIL2-4), as depicted for 30 hai in Figure 3a and for 50 hai in Figure 3b). Potentially, the activity of both QTL leads to the (stacked) activation of multiple WRKY genes. In contrast to the NBS-LRR and glucanase gene families we found relatively less WRKY genes differentially expressed at 50 hai: No more than 20% of the 116 identified WRKY genes were differentially expressed for any genotype.

**Figure 4:**
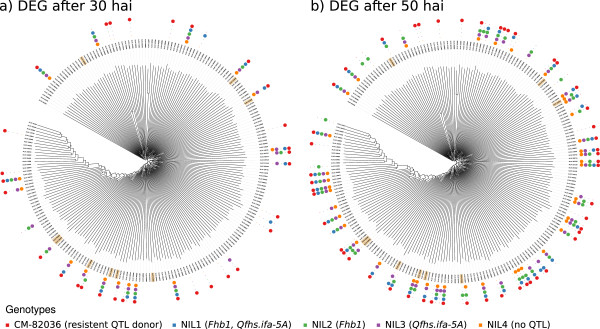
**Regulation of UGT genes at different time points.** Dendrograms display differential expression of UGT genes **(a)** 30 and **(b)** 50 hours after inoculation (hai) with *F. graminearum* spores. Genes that are significantly changed for the given genetic background (NIL1-NIL4, CM-82036) in response to *F. graminearum* are indicated in the respective colour. UGT genes homologous to the previously identified DON-detoxification UGTs in *B. distachyon* are highlighted. Note that the clades within dendrogram do not necessarily reflect groups or families of related genes, but are only used for presentation purposes.

UGTs have been shown to encode the ability to inactivate the *F. graminearum* toxin DON by formation of DON-3-glucoside in *Arabidopsis thaliana* Heynh. (D3G, [44]) and such genes also exist in monocotyledoneous species, where they are specifically upregulated in response to the toxin [41,45]. Our analysis found relatively few UGTs responsive to *F. graminearum* compared to the total of 222 identified putative UGTs (Figure 4). We found no specific upregulation of UGTs in lines sharing *Fhb1*, the QTL associated with detoxification by glycoconjugation of the *F. graminearum* toxin DON ([11]). However, 50 hai, besides CM-82036 (27 differential expressed genes), NIL2, harboring *Fhb1* only, exhibited the highest number of significantly changed UGTs (24). Also, while most of the DEG were found in more than just one NIL, 6 UGTs are only differentially expressed for NIL2. Recently, we have characterized a monocotyledonous UGT gene family, which encodes isozymes capable of inactivating the toxin [41]. We have identified nine putative orthologs to this gene family in our data (highlighted in Figure 4a and 4b) of which 2 were again specifically changed only for NIL2 at 50 hai (Figure 4b), while the others were either not differentially expressed at all (3) or upregulated in more than one genotype without exhibiting any QTL dependent patterns (4).

## Discussion

The defense response of wheat to one of its most devastating pathogens *F. graminearum* has been investigated in multiple transcriptome profiling studies, which compared differentially resistant genotypes [46-48] or reported on the specific response to DON [49-51]. The general understanding of the wheat/*F. graminearum* interaction has been further expanded by proteomic and metabolomic studies [12,52]. Statistics on large-scale data often rely on the detection of isolated significantly changed genes. These genes represent only a fraction of the entire defense response and potentially might not include the causative gene for mounting the resistant reaction. Here we present a co-expressed gene network, which enabled us to detect gene modules that are active in the susceptible and/or resistant genotypes. A network-driven approach has the advantage of describing coordinated gene expression changes in a holistic manner [53]. The interaction information can then be analyzed using mathematical approaches that can select features of interest for the subsequent analyses, e.g. [54,55]. Integration of DEG information into the gene co-expression network allowed for observing genotype specific dynamics in the response. In the work at hand we analyzed the effect of two major QTL by analyzing gene expression profiles of NILs segregating for either QTL. The identified modules comprise groups of genes that act in concerted manner in reaction to the pathogen – and in part are also specific for genotype or the QTL. It needs however to be noted that these effects may also be influenced by the activity of closely linked, yet QTL independent genes, that have been introgressed with the QTL during the generation of the near-isogenic material. To understand the functional background of these modules it will be necessary to combine information about the genes in these modules and their interactions with additional data, which will be part of future studies. The publication of the bread wheat genome will contribute to this challenge by allowing to further split the network into subgenome-specific partitions.

The time points we chose to collect samples after inoculation with *F. graminearum* reflect crucial stages of the initial biotrophic growth phase of *F. graminearum* (30 hai) and in the onset of necrotrophic growth (50 hai): Germination and hyphae development occurs within 24 hai and the formation of infection hyphae has been observed at 36 hai [56]. The colonization of cells and spread into rachis and adjacent spikelet is reported to occur at about 48 hai [57]. Significant levels of DON were only found after this time point [14,46]. We observed two distinct defense-related modules in the gene co-expression network, which showed a time point dependent enrichment with DEG. Module B, comprising well over 1,000 genes in our network, was significantly enriched for genes upregulated in presence of the pathogen already at 30 hai and more so at 50 hai. In contrast module G was significantly enriched for DEG only at 50 hai. Potentially, module G could reflect reaction to the transformation of the fungus into the necrotrophic stage and possibly to DON, which is not likely to be present earlier. Module G was higher enriched for the more susceptible genotypes NIL2 (*Fhb1*) and NIL4 (no QTL) but also for CM-82036. NIL2 and NIL4 lack the resistance against initial infection conferred by *Qfhs.ifa-5A* and consequently a higher infection rate could have elicited a stronger response in these genotypes. The enrichment for CM-82036 may correspond to a general faster induction of defense mechanisms.

### Activation of glycolysis and amino acid biosynthesis in response to *F. graminearum*

Our analysis finds genes involved in the pentose phosphate pathway and citric acid cycle upreguated in response to the pathogen in all genotypes. Additional terms for the pentose phosphate pathway and also for the glutathione-mediated detoxification of the toxic respiration biproduct methylglyoxal (glyoxylase I, [58]) have been found for NIL3. These findings demonstrate the elevated demand of carbohydrates and energy equivalents during the resistance response and also the probable breakdown of photosynthesis, which is required to replenish energy equivalents from carbohydrates (reviewed in [28]). The glycolysis-generated NADPH could also be used to fuel the production of reactive oxygen species. However, we do not find terms for the central enzyme in production of reactive oxygen species (ROS), NADPH oxidase in our analysis. Acquisition of the required hexoses heavily relies on the activity of cell wall invertases, which have also not been detected in our analysis. Transient silencing of a tobacco invertase, severely reduces the expression of defense-related genes [59]. However, invertase activity might not be sufficient to meet the increased requirements. The citric acid cycle can be replenished via the GABA-shunt, which utilizes glutamate as substrate [29]. We find GO terms for glutamate synthases and glutamine metabolic processes abundant already 30 hai, which could be upregulated to support the GABA-shunt. An alternative explanation suggests an indirect role in the production of secondary metabolites: Glutamine synthase have been shown upregulated in concert with phenylalanine ammonia lyases (PAL), which catalyse the transformation of phenylalanine to trans-cinnamate and represent the first dedicated step in the biosynthesis of phenylpropanoids and lignin. Ammonium is a side product of this process and may be reutilized by glutamine synthases also in order to prevent the accumulation to toxic levels.

### The resistant CM-82036 exerts its successful defense by reacting earlier and with a specific subnetwork

The unique response of CM-82036 was already reflected in the high number of DEG at 30 hai in comparison to the four NILs. Also for CM-82036 a much higher number of glucanases, NBS-LRR and WRKY genes were activated earlier. The faster response in comparison to susceptible genotypes has been previously observed [57]. Not only transcript levels of putative resistance genes but also faster induction of such genes seem to be a decisive factor in mounting a successful defense response [48,52]. What distinguished the response of CM-82036 was also a unique response module (Figure 2 Module H), which was not observed for any of the other lines. Besides genes involved in signaling and control of gene expression GO annotations of this module highlight terms for terpenoid and trehalose biosynthesis at 30 hai. The role of the disaccharide trehalose in plant defense has been recently reviewed [60]. Trehalose has been reported as a ROS quencher in wheat [61] and induces the expression of a WRKY6 gene and a glucanase gene in *A. thaliana*[28]. Treatment with trehalose confers partial protection against *Blumeria graminis* Speer to wheat [62]. The present study also found a considerable higher number of glucanase genes significantly changed for the earlier time point compared to the other investigated genotypes, which could relate to trehalose activity. However, it remains unclear as how the sugar exerts these functions.

### A diacylglycerol kinase and early induction of JA and ET biosynthesis are associated with lines harboring *Fhb1*

We have observed effects of *Fhb1* in different genetic backgrounds by identifying *F. graminearum* responsive transcripts that are changed only for lines harboring the resistant *Fhb1* allele. Among the few shared transcripts we found terms for G protein coupled signaling and diacylglycerol kinase activity (describing the same gene, 30 and 50 hai). Loss of G protein dependent phosphatidic acid signaling leads to reduced accumulation of defense-associated transcripts: Plant G proteins act in reception and translation of extracellular cues into intracellular second messengers. In rice the expression of the G protein α subunit *RGA1* is R-gene dependent and *rga1* mutants show a delayed production of ROS in response to *Magnaporthe grisea* Barr elicitors [63]. The same authors could later show that a mitogen-activated protein kinase is the downstream target of RGA1. Silencing this kinase leads to reduced levels of PR proteins and PAL [64]. A role for *phosphatidic* acid signaling in *F. graminearum* resistance was previously suggested by Ding and associates [52]. In this proteomic/transcriptomic study the authors find among other transcripts also diacylglycerol kinase and phospholipase D less abundant in a *F. graminearum*-susceptible mutant of the *Fhb1*-harboring resistant line Wangshuibai. Also in *A. thaliana* mutants of the G protein β subunit ABG1 were shown more susceptible to a variety of fungal pathogens, including *Fusarium oxysporum* Schltdl. [65].

The expression of *Fhb1*-related genes could be coupled to disease development/presence of DON and hence to the overall resistance conferred by the respective genotype, which may delay disease development to a certain extent. The lines containing *Fbh1* exhibit broad differences in FHB resistance: The highly resistant CM-82036 develops disease symptoms exclusively on the infected spikelets, while NIL1 (both QTL) and NIL2 (only *Fhb1*) exhibit just an intermediate level of resistance [14]. Consequently, *Fhb1*-associated transcripts are not necessarily significantly changed for all these lines at a given time point. To reduce complexity, we have also investigated DEG for NIL2 only, which harbors *Fhb1* in a susceptible background. DEG encoded for proteins involved in the biosynthesis of biotic stress response hormones JA and ET already at 30 hai. Both have been implicated with resistance mediated by the QTL donor line Sumai-3 [66]. Recent work using Virus-induced gene silencing in wheat showed that plants impaired in the production of ET are more susceptible to the disease [67]. JA has also received some attention in respect of *Fhb1* recently: The QTL was associated with a higher abundance of JA in a proteomic study using a NIL pair differing in *Fhb1*[50]. In the first transcriptome sequencing study investigating an *Fhb1*-deletion line of Wangshuibai, the authors find no difference in the abundance of transcripts corresponding to JA biosynthesis genes, but hypothesize that JA signaling in the deletion line is impaired, since downstream targets of JA are induced in the wildtype but not in the deletion line [61]. These findings may be related to the *Fhb1*-associated G protein coupled kinase, which could be involved in transmitting JA signals.

*Fhb1* confers resistance against spreading of the disease [9]. The QTL also co-localizes with resistance against DON – a virulence factor for *F. graminearum*[11] and it is very likely that both QTL relate to the same causal gene. Consequently, the QTL may exert its function only in the necrotrophic growth phase of the fungus. The resistant response in NIL2 appears delayed when observing dynamics of gene expression for four closer characterized gene families (Figures 3 and 4, Additional files 16 and 17). While CM-82036 exhibits the highest number of DEG at 30 hai for glucanase, NBS-LRR, WRKY and UGT genes, NIL2 and NIL4 show fewer DEG than the other lines at this time point. At 50 hai many more genes are upregulated for all genotypes (Table 2). By then, NIL2 has caught up and exhibits a similar strong response as CM-82036, while NIL4 still contains among the lowest number of DEG. The stronger response for NIL2 at 50 hai, could suggest the requirement of additional environmental cues such as penetration of host tissue/DON accumulation, which may not be present at 30 hai. Especially the UGT gene family is interesting in respect of *Fhb1*: Lines harboring the QTL contain a higher ratio of the UGT-mediated DON detoxification product DON-3-glucoside [11]. Wheat UGTs orthologous to a recently described gene cluster of *B. distachyon*[41] harboring DON-detoxification UGTs potentially share this ability. Our analysis identified 9 close homologs, which are likely candidates for future functional analysis. 2 and 7 of these homologs are expressed 30 and 50 hai, respectively, which is in line with the observation that these UGTs are not induced by *F. graminearum* but specifically for the toxin [41]. However, only few genes are specifically induced in lines with *Fhb1* or NIL2 only.

### Lines harboring *Qfhs.ifa-5A* exhibited higher activity in defense module B-sub2 and were associated with calcium signaling and riboflavin biosynthesis

In contrast to *Fhb1*, *Qfhs.ifa-5A* confers type I resistance against initial infection of *F. graminearum*[9]. Although the infection method used in this study favors the phenotypic assessment of type II resistance, the resistance mediated by *Qfhs.ifa-5A* can be assessed using this technique, and consequently also the QTL-specific transcriptional response may be captured. In this study we found more genes in the defense-associated network module B-sub2 differentially expressed for lines harboring *Qfhs.ifa-5A* at 30 hai (CM-82036, NIL1, NIL3) than for lines without the QTL. While this may not represent a QTL-specific gene subnetwork, we suggest that these genes were faster or stronger differentially expressed for these lines due to the activity of the *Qfhs.ifa-5A*. We identified a group of central genes encoding protein kinases and a CYP gene within this module, which were earlier induced for *Qfhs.ifa-5A* lines. These all are likely candidates for future functional analysis. Submodule B-sub2 comprised 397 genes and included kinases activity, glutamate-gated ion channels and genes involved in tRNA processing (Additional file 13). Elevated tRNA abundance has been previously linked to the response to DON in barley [68] and other abiotic stresses [69]. Higher translational activity could be a secondary effect to the toxin and not an active resistance response. Since, tRNA related terms are only found in the core set of DEG shared by all genotypes (Figure 1), we conclude that these most likely are not related to the resistance conferred by *Qfhs.ifa-5A*. In contrast, the glutamate-gated ion channel identified in the submodule was also changed only for lines sharing the QTL in the DEG analysis at 30 hai. Endogenous or environmental factors trigger changes in apoplasmatic glutamate concentration, which leads to the activation of these channels and subsequently to an intracellular increase of Ca^2+^[70]. Ca^2+^ influx is associated with early defense signalling [71]. Overexpression of ionotrophic glutamate receptors in *A. thaliana* leads to an increase in Ca^2+^ influx and consequently to a delayed infection with *Botrytis cinerea* Pers. [72]. Downstream targets of Ca^2+^ signaling such as ATPases or calmodulin are frequently reported as induced by *F. graminearum* (e.g. [52]). Genotypes harboring *Qfhs.ifa-5A* also share GO terms relating to the biosynthesis and the metabolism of riboflavin (30 hai). Riboflavin has been reported to induce resistance to fungal and other pathogens [73], potentially by recruiting NPR1, the essential regulator of systemic acquired resistance, independently from the defense-signaling hormone salicylic acid, which is strongly associated with NPR1 [74]. Riboflavin is also implicated in the activation of ethylene biosynthesis [73] and we also find genes related to ethylene biosynthesis in the section shared by lines harboring *Qfhs.ifa-5A* (Additional file 6, 30 hai).

Taken together this study provides insights into resistance response of differentially resistant wheat genotypes to *F. graminearum*. By combining a gene co-expression network approach with differential gene expression analysis we were able to make observation of genes and pathways associated with two prominent resistance QTL, *Fhb1* and *Qfhs.ifa-5A*. Central genes within the network may be valid candidate genes for functional testing.

## Conclusions

This RNA-seq study provides insights into the QTL-dependent defense response of bread wheat against *F. graminearum*. We find G-protein coupled receptor kinases and biosynthesis genes for jasmonate and ethylene earlier induced for NILs harboring *Fhb1* and genes involved in the biosynthesis and metabolism of riboflavin were found more abundant after infection in lines harboring *Qfhs.ifa-5A*. By combining a gene co-expression network approach with differential gene expression analysis we identified genes and pathways associated with the investigated NILs and the resistant parent CM-82036. Central genes within the network may be promising candidate genes for functional testing. Revisiting these and other data after the complete wheat genes are available will provide even higher resolved insights into the defense response dynamics within the gene co-expression network.

## Methods

### Plant material and inoculation experiment

Four NILs previously generated from a cross of the resistant spring wheat line CM-82036 and Remus, a susceptible German spring wheat cultivar [14] were investigated in these experiments and also the resistant parent CM-82036. The NILs have been developed from one BC5F1 plant with Remus as the recurrent parent (5 backcrosses). In the BC5F2 lines that contain the resistance alleles from CM-82036 of both *Fhb1* and *Qfhs.ifa-5A* (NIL1), or either *Fhb1* (NIL2) or *Qfhs.ifa-5A* (NIL3) or none (NIL4) have been selected. *F. graminearum* conidia spores required for inoculation were produced on defined SNA medium under UV-light at 25°C. After two weeks conidia were harvested and diluted to 50,000 conidia/mL in water. Aliquots were stored at −80°C [10].

Plant growth conditions and the inoculation of flowering plants with *F. graminearum* spores were described previously [14]. Briefly, 12 florets per head (from 6 central spikelets, the two basal florets) were inoculated at anthesis with 10 μl of a *F. graminearum* conidia spore suspension (500 conidia, concentration 50.000 conidia / mL) or mock by cautiously inserting a droplet onto the generative part of each floret without wounding the tissue. The treated heads were moistened with water and covered in plastic bags for 24 hours to provide humid conditions favorable for infection with the pathogen. Only palea and lemma of the inoculated florets were sampled including the respective part of the rachis. For each of the 60 samples (five genotypes, *F. graminearum*/mock treatment, two time points 30 and 50 hai, three replicates) 12 heads were used and pooled into one combined sample and stored at −80°C until use.

### RNA-extraction and sequencing

To eliminate RNases, metal jars with inherent metal spheres for Retsch-mill (MM 301, Haan, Germany) were sterilized at 180°C for 3 h and then stored at −80°C. All tissue belonging to one sample was pooled in one precooled jar and clamped in Retsch-mill. Grinding was performed for 30 seconds at full speed to obtain a fine tissue powder and immediately put back at −80°C. Total-RNA was extracted from 100 mg of frozen tissue powder using the RNeasy Plant Mini Kit (#74903, Qiagen, Venlo, Netherlands) according to manufacturer’s instructions. The extracted RNA was checked for quality and quantity on an automated electrophoresis-system (Experion, #701-7000, Bio-Rad, Hercules, CA, US). Sequencing was performed on an Illumina HiSeq2000 machine using 8x multiplexing, theoretically generating 22 M reads per sample by the sequencing-provider GATC (Konstanz, Germany). The respective data sets are available in the EBI ArrayExpress (http://www.ebi.ac.uk/arrayexpress/) repository under the accession number E-MTAB-1729.

### Data processing and mapping

The recently published LCG wheat assembly [5] was used to identify RNA-Seq transcripts with Tophat and Cufflinks [24]. We removed one mock-treatment sample from genotype NIL2 (50 hai) as it did not pass quality control. Transcripts were combined with Cuffcompare and were mapped against barley high confidence genes [18] with Vmatch (http://www.vmatch.de) (exdro*p =* 3, seedlength = 12, hit length = 100 bp, identity > 85%) requiring a BBH and by taking the longest transcript of each reported gene loci. Transcripts with existing BBH to a barley gene served as input for network analysis and enrichment analyses.

### Statistical analysis of differential gene expression

Differentially expressed genes (DEG) were detected with Cuffdiff [24] between treatments in pair-wise comparisons. These were performed for samples from the same genotype but for different treatments or time-points. Thus we compared mock-inoculation against Fusarium-inoculation (M/F) at 30 hai and 50 hai. Additionally, we compared mock-inoculation at 30 hai with 50 hai (M/M). Cuffdiff default parameters were applied and the FDR-adjusted *p*-value was taken as a cut-off, keeping values below 0.1 as DEG. Genes that were significantly differentially expressed in M/M were not considered as differentially expressed in M/F comparison and *vice versa*.

### Co-expression network and module detection

Gene expression values (FPKM) were taken to infer a gene co-expression network. We used the log_2_-transformed FPKM values, and replaced values smaller than one by zero. In order to keep only the most active genes we applied a coefficient of variation filter with a threshold of 1 across the different conditions. In order to infer a network, we made use of the WGCNA package [29] with the soft-thresholding parameter beta set to 4 and absolute Pearson’s correlation coefficient. Thereby, we inferred an undirected, weighted network. Within the network, clusters of genes with similar expression patterns, so called modules, were then inferred using a clustering of the Topological Overlay Matrix (cutreeDynamic method; deepSplit = 2, minimal module size = 40, merging similar modules with parameter cut height = 0.2). The module eigengene (ME, [30]) of a given module is defined by the first principal component of the module expression matrix and can be regarded as the representative of the gene expression in a module [31]. By using the ME we could quantify the association between a module and the samples, with larger values indicating a stronger association with a module.

### GO and interpro enrichment analyses

GO terms and Interpro domains for the BBH transcripts were extracted from the barley repository (ftp://ftpmips.gsf.de/plants/barley/public_data/). GO enrichment analyses were performed using the topGO package [26], using Fisher’s exact test. To reduce the number of false positive findings the *elim* algorithm was applied and reported *p*-values smaller than 0.05 were kept for further analyses. Interpro terms were tested for enrichment using one-sided Fisher’s exact test, keeping Benjamini-Hochberg [25] adjusted *p*-values smaller than 0.05.

## Competing interests

The authors declare no competing interests.

## Authors’ contributions

KK, KM, WS, HB designed the research; KK, GS, TN, BS, ML performed the research; KK, TN, CA, MS, WS analyzed the data; KK, GS, TN, WS wrote the paper. All authors read and approved the final manuscript.

## Supplementary Material

Additional file 1 **Raw read counts per sample.** The number of mapped reads per sample. Click here for file

Additional file 2 **Quality and Coverage of RNA-seq reads.** Coverage of RNA-seq along published wheat fl-cDNA. Click here for file

Additional file 3 **Fusarium graminearum mapped read counts.** Mapping of RNA-seq against *Fusarium graminearum* genes. Click here for file

Additional file 4 **Comparison of Fusarium graminearum mapped reads.** Statistical overview of mapped reads against Fusarium graminearum per sample and condition. Click here for file

Additional file 5 **List of differentially expressed genes.** A summary of differentialy expressed genes. Click here for file

Additional file 6 **Enrichment analyses of DEG at 30 hai.** Results of GO and Interpro enrichment analyses for DEG at 30 hai. Click here for file

Additional file 7 **Enrichment analyses of DEG at 50 hai.** Results of GO and Interpro enrichment analyses for DEG at 50 hai. Click here for file

Additional file 8 **Module inference.** Colored dendrogram showing the modules as inferred from the co-expression network. Click here for file

Additional file 9 **Gene module membership.** The module membership of the analyzed genes as well as the contig mapping information. Click here for file

Additional file 10 **Module eigengenes.** Summary of the module eigengenes. Click here for file

Additional file 11 **Enrichment analyses for modules.** Results of GO and Interpro enrichment analyses for modules B, G, and H. Click here for file

Additional file 12 **DEG in submodules of module B.** Similar to Figure 2 the ratio of DEG in the submodules of module B is depicted. Click here for file

Additional file 13 **Enrichment analyses for modules of module B.** Results of GO and Interpro enrichment analyses for module B submodules 1 and 2. Click here for file

Additional file 14 **Enrichment analyses for central genes.** Results of GO and Interpro enrichment analyses for central genes. Click here for file

Additional file 15 **Differentially expressed central genes.** Differentially expressed central genes and annotations with frequently observed GO terms. Click here for file

Additional file 16 **Regulation of NBS-LRR genes at different time points.** The line-specific regulation of NBS-LRR genes at different time points. Click here for file

Additional file 17 **Regulation of glucanases at different time points.** The line-specific regulation of glucanases at different time points. Click here for file

## Supporting data

The data set supporting the results of this article is available in the ArrayExpress repository (http://www.ebi.ac.uk/arrayexpress), accession number E-MTAB-1729.
