# Supplementary material for: Quantitative trait loci-dependent analysis of a gene co-expression network associated with Fusarium head blight resistance in bread wheat (Triticum aestivum L.)
Source: BMC Genomics. 2013 Oct 24;14:728. doi: 10.1186/1471-2164-14-728 (PMC4007557; doi:10.1186/1471-2164-14-728)
Supplement: Additional file 1 — Raw read counts per sample. The number of mapped reads per sample. [file 1471-2164-14-728-S1.docx]

**Additional File 1 – Raw read counts per sample**The number of mapped reads per sample

| **sample** | **Reads total  replicate 1** | **Reads total**  **replicate 2** | **Reads total**  **replicate 3** |
| --- | --- | --- | --- |
| **NIL1:F30** | 24.6 | 29.2 | 23.6 |
| **NIL1:F50** | 30.5 | 35.4 | 31.3 |
| **NIL1:M30** | 24.8 | 36.6 | 30.2 |
| **NIL1:M50** | 40.1 | 33.2 | 21.4 |
| **NIL2:F30** | 26.2 | 37.7 | 27.2 |
| **NIL2:F50** | 43.5 | 28.1 | 31.5 |
| **NIL2:M30** | 33.7 | 27.4 | 23.4 |
| **NIL2:M50** | 34.4 | 44.9 | 27.2 |
| **NIL3:F30** | 23.3 | 37.0 | 45.1 |
| **NIL3:F50** | 30.3 | 27.1 | 32.6 |
| **NIL3:M30** | 31.7 | 32.1 | 30.2 |
| **NIL3:M50** | 28.3 | 42.9 | 27.6 |
| **NIL4:F30** | 32.8 | 32.9 | 35.4 |
| **NIL4:F50** | 27.3 | 20.3 | 33.4 |
| **NIL4:M30** | 32.4 | 30.7 | 27.6 |
| **NIL4:M50** | 23.1 | 23.8 | 36.5 |
| **CM-82036:F30** | 28.6 | 22.2 | 44.7 |
| **CM-82036:F50** | 29.6 | 20.6 | 27.0 |
| **CM-82036:M30** | 30.6 | 27.1 | 21.7 |
| **CM-82036:M50** | 30.1 | 22.3 | 32.3 |

Numbers reflect million reads. F30: Fusarium-inoculation at 30 hours after inoculation (hai); F50: Fusarium-inoculation at 50 hai; M30: mock-inoculation at 30 hai; M50: mock-inoculation at 50 hai
