# Supplementary material for: Quantitative trait loci-dependent analysis of a gene co-expression network associated with Fusarium head blight resistance in bread wheat (Triticum aestivum L.)
Source: BMC Genomics. 2013 Oct 24;14:728. doi: 10.1186/1471-2164-14-728 (PMC4007557; doi:10.1186/1471-2164-14-728)
Supplement: Additional file 2 — Quality and Coverage of RNA-seq reads. Coverage of RNA-seq along published wheat fl-cDNA. [file 1471-2164-14-728-S2.docx]

**Additional File 2 – Quality and Coverage of RNA-seq reads**Coverage of RNA-seq along published wheat fl-cDNA.


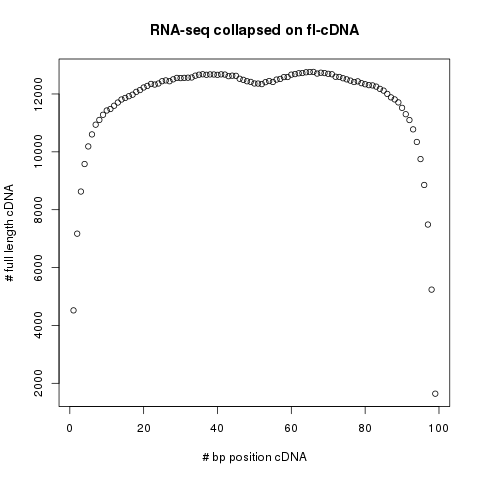


RNA-seq reads were mapped on public wheat fl-cDNA [37]. X-axis reflects sequence length in percent. The amount of fl-cDNA with RNA-seq support.
