# Supplementary material for: Quantitative trait loci-dependent analysis of a gene co-expression network associated with Fusarium head blight resistance in bread wheat (Triticum aestivum L.)
Source: BMC Genomics. 2013 Oct 24;14:728. doi: 10.1186/1471-2164-14-728 (PMC4007557; doi:10.1186/1471-2164-14-728)
Supplement: Additional file 3 — Fusarium graminearum mapped read counts. Mapping of RNA-seq against Fusarium graminearum genes. [file 1471-2164-14-728-S3.docx]

**Additional File 3 – Fusarium graminearum mapped read counts**Mapping of RNA-seq against *Fusarium graminearum* genes.

| **sample** | **Reads total replicate 1** | **Reads total replicate 2** | **Reads total replicate 3** |
| --- | --- | --- | --- |
| **NIL1:F30** | 69,725 | 118,454 | 32,260 |
| **NIL1:F50** | 95,490 | 101,684 | 70,703 |
| **NIL2:F30** | 49,175 | 89,139 | 41,743 |
| **NIL2:F50** | 149,825 | 136,224 | 76,102 |
| **NIL3:F30** | 40,490 | 90,163 | 103,416 |
| **NIL3:F50** | 69,240 | 92,497 | 81,557 |
| **NIL4:F30** | 64,507 | 83,817 | 61,041 |
| **NIL4:F50** | 75,027 | 76,895 | 85,719 |
| **CM-82036:F30** | 86,054 | 52,784 | 135,042 |
| **CM-82036:F50** | 167,337 | 86,168 | 74,795 |
| **NIL1:M30** | 1,113 | 1,564 | 1,577 |
| **NIL1:M50** | 1,533 | 1,837 | 981 |
| **NIL2:M30** | 1,421 | 2,123 | 1,502 |
| **NIL2:M50** | 1,452 | 1,713 | 10,698 |
| **NIL3:M30** | 1,527 | 1,103 | 2,458 |
| **NIL3:M50** | 1,531 | 1,810 | 1,327 |
| **NIL4:M30** | 1,776 | 1,153 | 2,205 |
| **NIL4:M50** | 585 | 577 | 1,707 |
| **CM-82036:M30** | 1,509 | 953 | 1,043 |
| **CM-82036:M50** | 1,003 | 527 | 1,276 |

Due to the relatively large number of mapped *F. graminearum* mapped reads in NIL2_M50_R3 we decided to exclude the replicate from further analyses. F30: Fusarium-inoculation at 30 hours after inoculation (hai); F50: Fusarium-inoculation at 50 hai; M30: mock-inoculation at 30 hai; M50: mock-inoculation at 50 hai.
