# Supplementary material for: Quantitative trait loci-dependent analysis of a gene co-expression network associated with Fusarium head blight resistance in bread wheat (Triticum aestivum L.)
Source: BMC Genomics. 2013 Oct 24;14:728. doi: 10.1186/1471-2164-14-728 (PMC4007557; doi:10.1186/1471-2164-14-728)
Supplement: Additional file 4 — Comparison of Fusarium graminearum mapped reads. Statistical overview of mapped reads against Fusarium graminearum per sample and condition. [file 1471-2164-14-728-S4.docx]

**Additional File 4 – Comparison of Fusarium graminearum mapped reads**Statistical overview of mapped reads against Fusarium graminearum per sample and condition.

|  | **Number of reads** | | | **Tested against** | | | |
| --- | --- | --- | --- | --- | --- | --- | --- |
|  | **Replicate 1** | **Replicate 2** | **Replicate 3** | **NIL2F30** | **NIL3F30** | **NIL4F30** | **CM-82036 F30** |
| **NIL1F30** | 69,725 | 118,454 | 32,260 | 0.370 | 0.904 | 0.859 | 0.749 |
| **NIL2F30** | 49,175 | 89,139 | 41,743 |  | 0.499 | 0.329 | 0.492 |
| **NIL3F30** | 40,490 | 90,163 | 103,416 |  |  | 0.710 | 0.656 |
| **NIL4F30** | 64,507 | 83,817 | 61,041 |  |  |  | 0.552 |
| **CM-82036 F30** | 86,054 | 52,784 | 135,042 |  |  |  |  |
|  |  |  |  |  |  |  |  |
|  | **Number of reads** | | | **Tested against** | | | |
|  | **Replicate 1** | **Replicate 2** | **Replicate 3** | **NIL2F30** | **NIL3F30** | **NIL4F30** | **CM-82036 F30** |
| **NIL1F50** | 95,490 | 101,684 | 70,703 | 0.158 | 0.525 | 0.508 | 0.526 |
| **NIL2F50** | 149,825 | 136,224 | 76,102 |  | 0.253 | 0.251 | 0.632 |
| **NIL3F50** | 69,240 | 92,497 | 81,557 |  |  | 0.810 | 0.502 |
| **NIL4F50** | 75,027 | 76,895 | 85,719 |  |  |  | 0.440 |
| **CM-82036 F50** | 167,337 | 86,168 | 74,795 |  |  |  |  |

Conditions were compared using t-tests. Given figures represent the p-values. F30: Fusarium-inoculation at 30 hours after inoculation (hai); F50: Fusarium-inoculation at 50 hai; M30: mock-inoculation at 30 hai; M50: mock-inoculation at 50 hai.
