# Supplementary material for: Quantitative trait loci-dependent analysis of a gene co-expression network associated with Fusarium head blight resistance in bread wheat (Triticum aestivum L.)
Source: BMC Genomics. 2013 Oct 24;14:728. doi: 10.1186/1471-2164-14-728 (PMC4007557; doi:10.1186/1471-2164-14-728)
Supplement: Additional file 8 — Module inference. Colored dendrogram showing the modules as inferred from the co-expression network. [file 1471-2164-14-728-S8.docx]

**Additional File 8 – Module inference**Colored dendrogram showing the modules as inferred from the co-expression network.


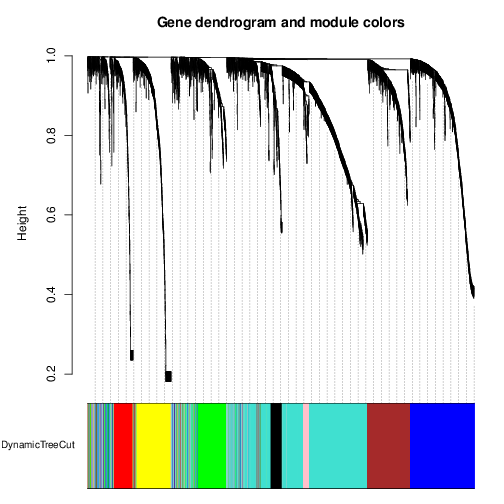


Colors represent the different modules.
