# Supplementary material for: Quantitative trait loci-dependent analysis of a gene co-expression network associated with Fusarium head blight resistance in bread wheat (Triticum aestivum L.)
Source: BMC Genomics. 2013 Oct 24;14:728. doi: 10.1186/1471-2164-14-728 (PMC4007557; doi:10.1186/1471-2164-14-728)
Supplement: Additional file 10 — Module eigengenes. Summary of the module eigengenes. [file 1471-2164-14-728-S10.docx]

**Additional File 10 – Module eigengenes**Summary of the module eigengenes.


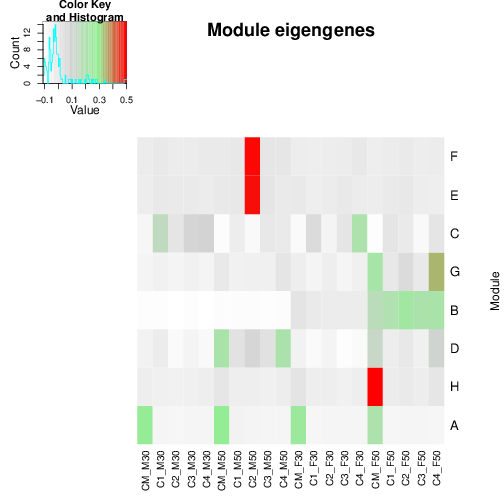


Module eigengenes (ME) as provided by the *moduleEigengenes* function in package WGCNA [Langfelder, Horvath, 2008] (averaged per condition).
