# Supplementary material for: Quantitative trait loci-dependent analysis of a gene co-expression network associated with Fusarium head blight resistance in bread wheat (Triticum aestivum L.)
Source: BMC Genomics. 2013 Oct 24;14:728. doi: 10.1186/1471-2164-14-728 (PMC4007557; doi:10.1186/1471-2164-14-728)
Supplement: Additional file 12 — DEG in submodules of module B. Similar to Figure 2 the ratio of DEG in the submodules of module B is depicted. [file 1471-2164-14-728-S12.docx]

**Additional File 12 – DEG in submodules of module B**Similar to Figure 2 the ratio of DEG in the submodules of module B is depicted.


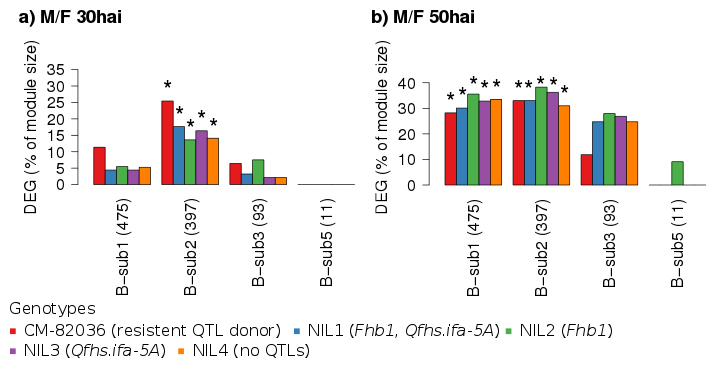


Number of DEG for each submodule with a ratio of differentially expressed genes (DEG) > 0.
* indicates a significant enrichment (one-sided Fisher's exact test; Bonferroni adjusted p-value < 0.05).
