# Supplementary figures and images for: Quantitative trait loci-dependent analysis of a gene co-expression network associated with Fusarium head blight resistance in bread wheat (Triticum aestivum L.)
Source: BMC Genomics. 2013 Oct 24;14:728. doi: 10.1186/1471-2164-14-728 (PMC4007557; doi:10.1186/1471-2164-14-728)

a) M/F 30 hai

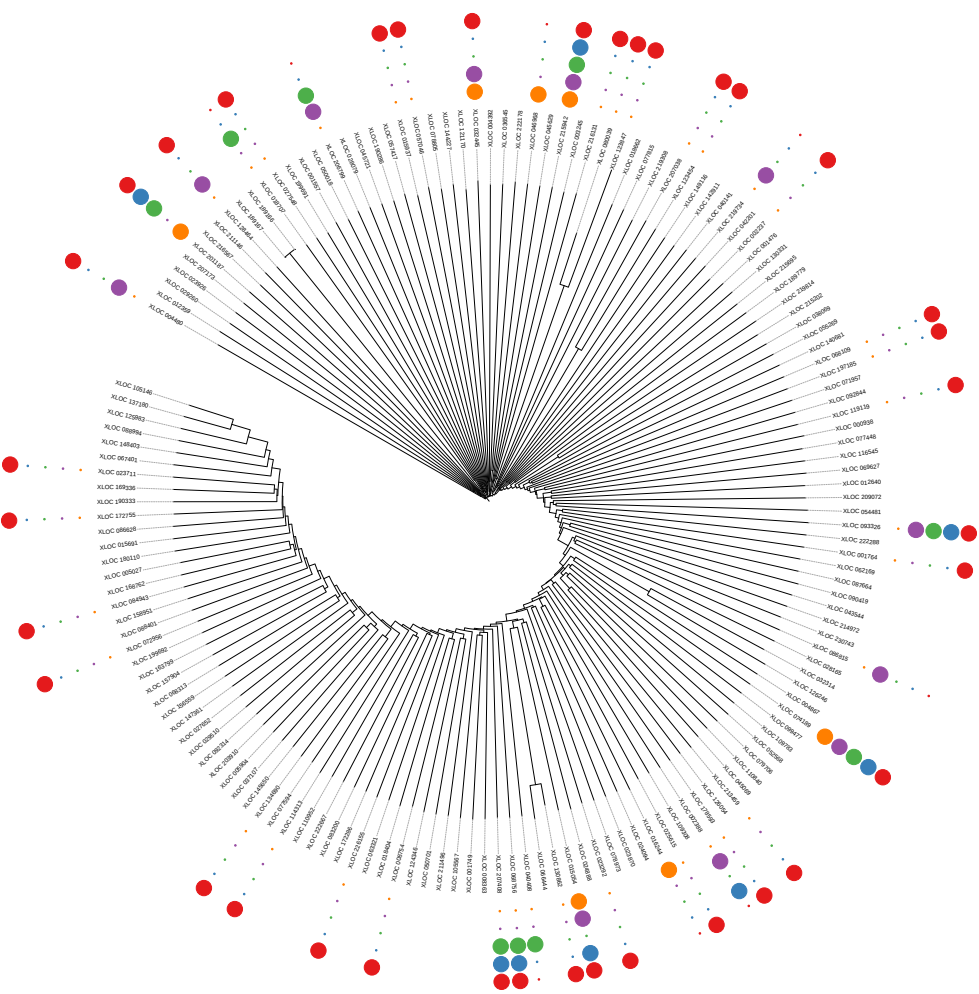

b) M/F 50 hai

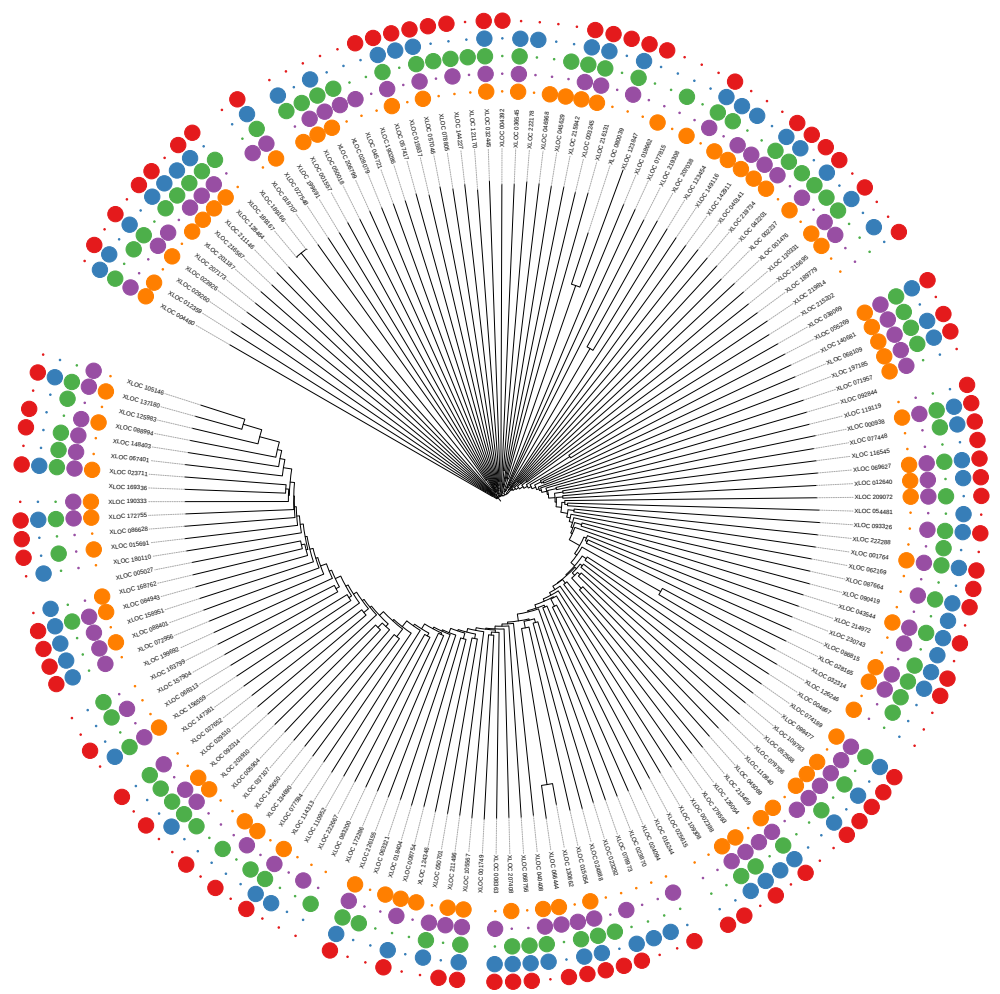

Genotypes

■ CM-82036 (resistant QTL donor) ■ NIL1 (*Fhb1*, *Qfhs.ifa-5A*) ■ NIL2 (*Fhb1*) ■ NIL3 (*Qfhs.ifa-5A*) ■ NIL4 (no QTL)

Supplement: Additional file 16 — Regulation of NBS-LRR genes at different time points. The line-specific regulation of NBS-LRR genes at different time points. [file 1471-2164-14-728-S16.pdf]
